# Secreted Protein Acidic and Rich in Cysteine (SPARC) Enhances Cell Proliferation, Migration, and Epithelial Mesenchymal Transition, and SPARC Expression is Associated with Tumor Grade in Head and Neck Cancer

**DOI:** 10.3390/ijms18071556

**Published:** 2017-07-18

**Authors:** Chih-Hau Chang, Meng-Chi Yen, Ssu-Hui Liao, Yu-Ling Hsu, Chung-Sheng Lai, Kao-Ping Chang, Ya-Ling Hsu

**Affiliations:** 1 Graduate Institute of Medicine, College of Medicine, Kaohsiung Medical University, Kaohsiung 807, Taiwan; igor8301023@gmail.com (C.-H.C.); s0970215575@gmail.com (S.-H.L.); l32861wa@yahoo.com.tw (Y.-L.H.); 2 Division of Plastic and Reconstructive Surgery, Kaohsiung Medical University Hospital, Kaohsiung 807, Taiwan; chshla@kmu.edu.tw (C.-S.L.); kapich@kmu.edu.tw (K.-P.C.); 3 Department of Emergency Medicine, Kaohsiung Medical University Hospital, Kaohsiung Medical University, Kaohsiung 807, Taiwan; yohoco@gmail.com; 4 Faculty of Medicine, College of Medicine, Kaohsiung Medical University, Kaohsiung 807, Taiwan

**Keywords:** secreted protein acidic and rich in cysteine, proliferation, migration, epithelial mesenchymal transition, head and neck cancer

## Abstract

Secreted protein acidic and rich in cysteine (SPARC) is a secreted protein which is involved in various biological processes. SPARC expression is associated with tumor metastasis and poor prognosis in several types of cancer. However, the SPARC-induced signaling pathway was not fully understood in head and neck cancer. In this study, our results showed that SPARC treatment promoted cell proliferation and migration in head and neck cancer cell lines FaDu and Detroit 562. In addition, SPARC induced expression of epithelial mesenchymal transition (EMT) regulators, including Slug, Snail, and Twist in Detroit 562. The results of phospho-kinase array analysis showed that SPARC treatment increased phosphorylation of some molecules including protein kinase B (PKB/AKT), ribosomal S6 kinase (RSK), and extracellular signal–regulated kinases (ERK). The expression of SPARC-induced EMT regulator Slug was suppressed by AKT inhibitor, but not ERK and RSK inhibitors. The SPARC expression in grade IV tumor samples is higher when compared to that in grade I–III tumor samples. Our results suggest that SPARC treatment enhances the EMT signaling pathway via activation of AKT, and exogenous SPARC and tumor expressing SPARC might be associated with tumor progression in head and neck cancers.

## 1. Introduction

Head and neck cancer is one of most common cancer types worldwide and every year an estimated 49,000 people are newly-diagnosed in United States [1]. The location of head and neck cancer includes paranasal sinuses, nasal and oral cavity, pharynx an dlarynx, and oral squamous cell carcinoma is the most common type among head and neck cancers [2]. Currently, smoking, alcohol, and human papillomavirus (HPV) infection are considered as risk factors of head and neck cancers [3,4]. However, the clinical prognosis of some patients is not fully explained by these risk factors. Therefore, some unknown factors which promote tumorigenesis in head and neck cancers need to be further investigated.

Recent evidence reveals that soluble factors in the tumor microenvironment results in immune escape, angiogenesis, proliferation, and epithelial to mesenchymal transition (EMT) properties in multiple types of cancers [5]. Cytokines, chemokines, and growth factors are key regulators of these signaling pathways [5,6]. Secreted protein acidic and rich in cysteine (SPARC, also known as osteonectin) is a glycoprotein which involve in various biological process [7]. It belongs to the matricellular protein family which regulates cell-matrix interactions and signaling pathways in cells [8]. SPARC is secreted from many types of cancer and tumor-associated stroma cells, and is considered to regulate tumor cell growth and metastasis [9]. However, the effect of SPARC reported in previous studies is different. In glioma and melanoma, the metastatic capacity is associated with SPARC expression [9]. SPARC promotes bone metastasis and EMT properties in highly-metastatic tumors, including triple-negative breast cancer, melanoma, and prostate cancers [10,11]. In contrast, low SPARC expression in cancer tissue is associated with good prognosis in patients with lung cancer and breast cancer [12,13]. The role of tumor-expressing SPARC is still paradoxical. Stromal cells-expressing SPARC also regulate the behavior of cancer cells and prognosis. Strong expression of SPARC in breast cancer stromal cells is correlated with good prognosis [14]. Another study indicates that stromal SPARC is pro-metastatic for breast cancer cells [15]. SPARC expression is frequently detected in tumor-associated stroma, but is not in pancreatic ductal adenocarcinoma. Stromal cells-expressing SPARC mediates invasiveness and metastatic capacity pancreatic cancer [16]. This evidence might suggest stromal SPARC is also a factor to induce tumor progression.

SPARC-related-signaling pathways and the role of exogenous and tumor-expressing SPARC have not been fully understood in head and neck cancers. In the present study, SPARC-induced phenotypes and signaling pathways in head and neck cancer cell lines were investigated and the expression of SPARC was examined in clinical tumor samples.

## 2. Results

### 2.1. Secreted Protein Acidic and Rich in Cysteine (SPARC) Treatment Enhances Cell Proliferation and Migration

**Figure 1:**
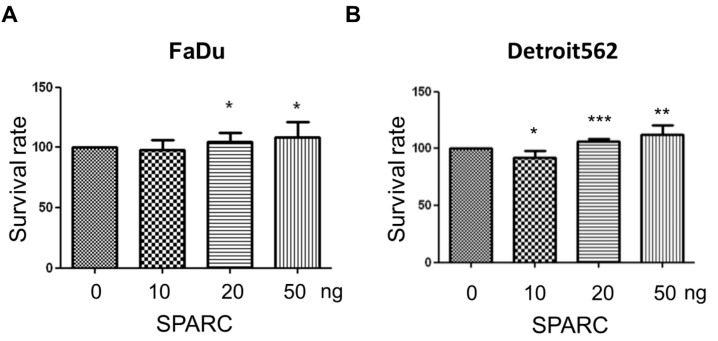
Secreted protein acidic and rich in cysteine (SPARC) treatment promoted cell proliferation. (**A**) FaDu and (**B**) Detroit 562. The error bars represent standard deviation (SD) (*t*-test; * *p* < 0.05, ** *p* < 0.01, *** *p* < 0.001, when compared with 0 ng group).

**Figure 2:**
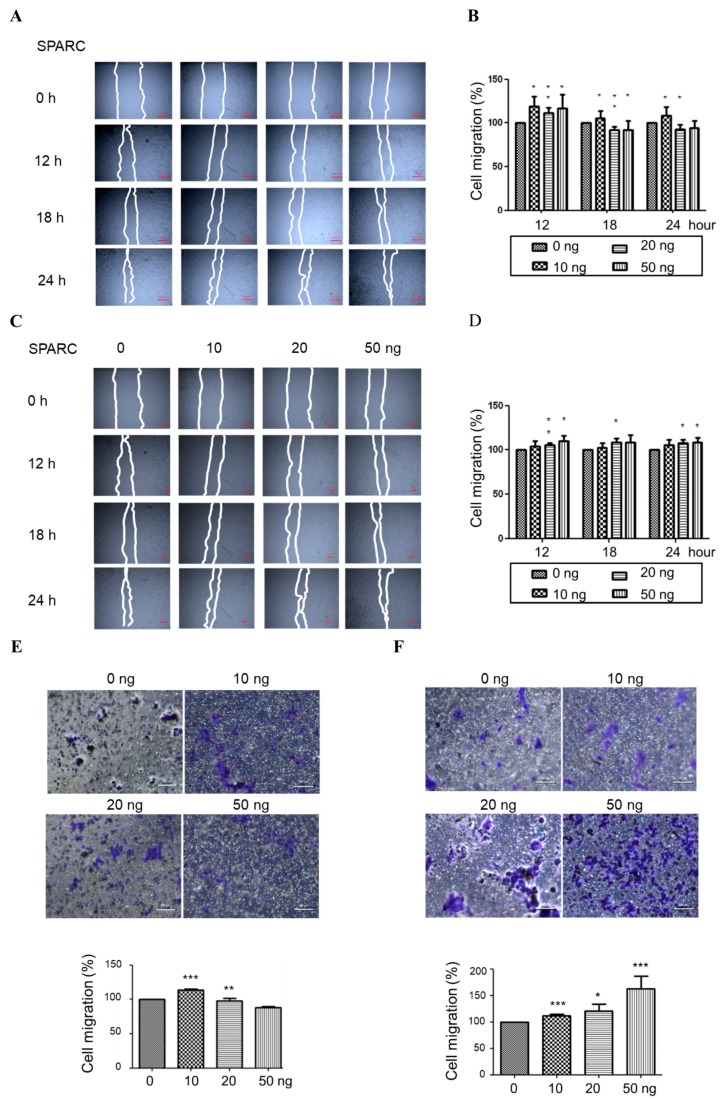
SPARC treatment promoted cell migration. (**A**) images of wound healing assay; (**B**) quantification of wound healing assay in FaDu cells; (**C**) images of wound healing assay; (**D**) quantification of wound healing assay in Detroit 562 cells; (**E**) transwell migration assay in FaDu cells and (**F**) Detroit 562 cells. Quantification results were shown in the lower panel and images were shown in the upper panel. The error bars represent SD (*t*-test; * *p* < 0.05, ** *p* < 0.01, *** *p* < 0.001, when compared with 0 ng group).

To investigate the effect of SPARC, the human pharyngeal carcinoma line Detroit 562, and human hypopharyngeal cancer cell line FaDu, were treated with different doses of recombinant human SPARC protein. The proliferation rate of Detroit 562 and FaDu was significantly induced (Figure 1A,B). In addition, our results showed that SPARC treatment enhanced migration capacity in wound-healing migration assay and transwell migration assay (Figure 2A–F). This suggests that SPARC was associated with malignant phenotypes in head and neck cancer cells.

### 2.2. SPARC Treatment Induces Epithelial Mesenchymal Transition (EMT) Phenotypes in Head and Neck Cancer Cells

**Figure 3:**
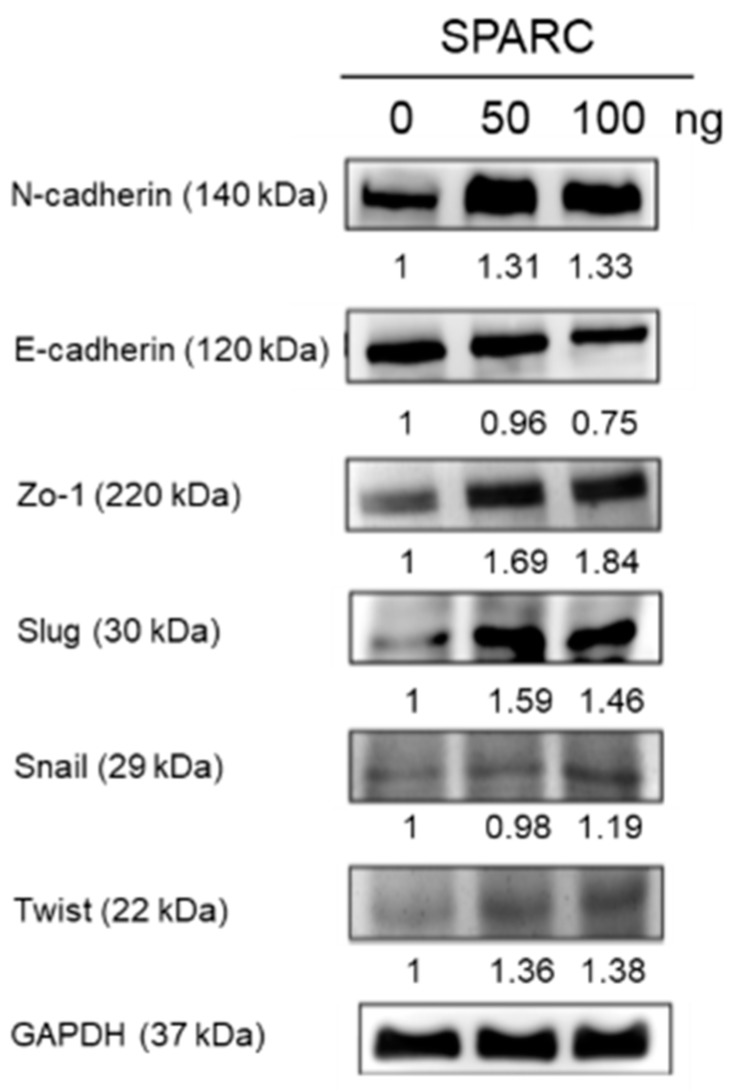
SPARC treatment promoted epithelial mesenchymal transition (EMT) signaling pathway: Western blot assay shows the protein expression levels of the EMT signaling pathways-related molecules. Band intensity was normalized to glyceraldehyde 3-phosphate dehydrogenase (GAPDH). Values are expressed relative to the control group (0 ng).

EMT changes the mobility of cells and is associated with tumor progression [17]. The process of EMT is regulated by various molecules including transcription factors Snail, Slug, and Twist [18]. When cells undergo EMT, decreasing epithelial cadherin (E-cadherin), and increasing neural cadherin (N-cadherin) [18]. Zo-1 is a tight junction protein and usually relocates from the membrane to the cytoplasm during EMT [19]. In Figure 3, the expression of N-cadherin, Zo-1, Slug, Snail, and Twist increased and the expression of E-cadherin slightly decreased in Detroit 562 cells after 50 and 100 ng SPARC treatment. The results suggest that SPARC treatment induced the mesenchymal phenotype in Detroit 562 cells.

### 2.3. Evaluation of SAPRC Treatment-Associated Kinases through Phosphor-Kinase Array

**Figure 4:**
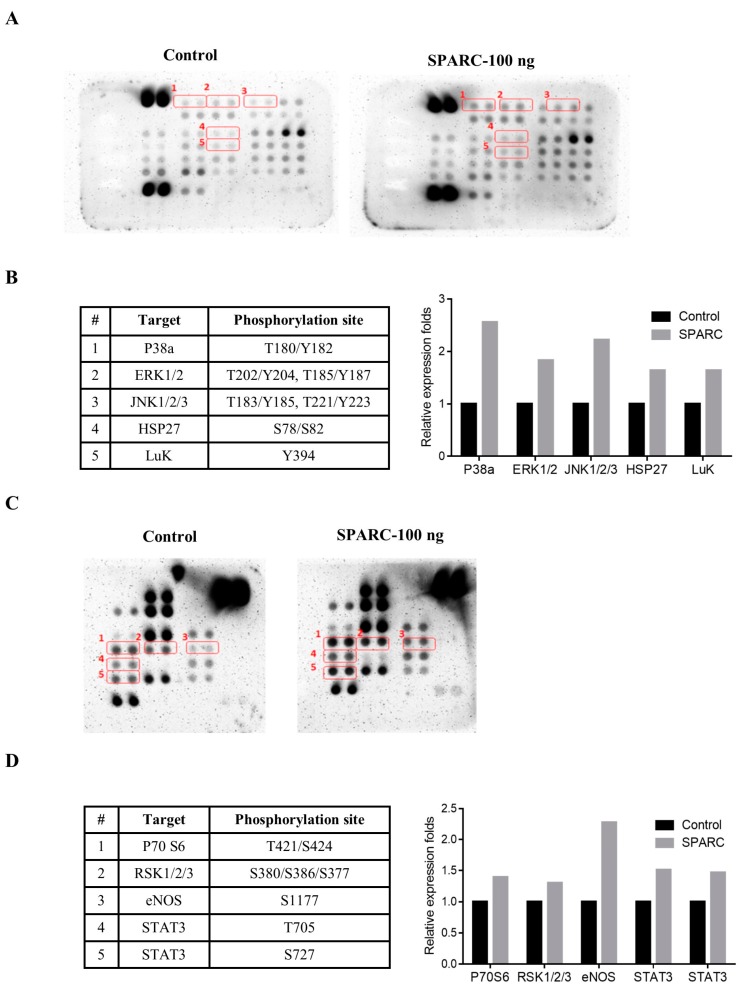
Investigation of SPARC treatment-activated signaling molecules by phospho-kinase array: 600 μg of protein extract of Detroit 562 cells were subjected to a set of phospho-kinase arrays (membrane A and B) in accordance with the manufacturer’s instructions. (**A**) Phospho-kinase array, membrane A the red frame which is respectively labeled number 1 to 5 indicate different phosphorylation site. The detail information is shown in Figure 4B; (**B**) the table in the left panel indicates the specific phosphorylation site of each molecule in membrane A; the quantification result (mean density value of two duplicate spots) is shown in the right panel. Values are expressed relative to the control group; (**C**) Membrane B, the red frame 1–5 indicates specific phosphorylation site in membrane B, and (**D**) the specific phosphorylation site of each molecule in membrane B and quantification result.

Currently, the SPARC-induced signaling pathways and -associated kinases are not well known in head and neck cancer. Thus, the phosphor-kinase array assay was performed. In Figure 4A–D, the phosphorylation of extracellular signal regulated kinase (ERK)1/2, p38a, jun amino-terminal kinases (JNK)1/2/3, heat shock protein (HSP) 27, Staphylococcal leucocidin (LUK), P70 S6, ribosomal S6 kinase (RSK)1/2/3, Endothelial NOS (eNOS), and signal transducer and activator of transcription 3 (STAT3) increased in Detroit 562 with 100 ng SPARC treatment and incubation for 24 h. We further confirmed the phosphorylated status of these proteins and SPARC-mediated signaling pathways through Western blot assay.

### 2.4. Investigation of SPARC Treatment Induced-Signaling Pathways

**Figure 5:**
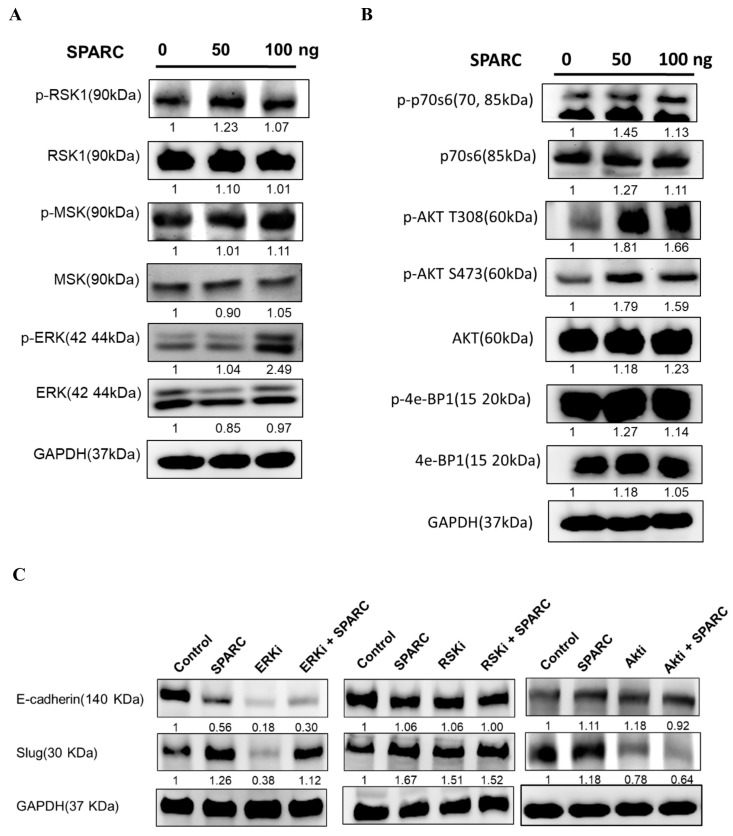
Investigation of SPARC treatment-induced signaling pathways by Western blot: Detroit 562 cells were treated with SPARC for 1.5 h and the protein extract was subjected to Western blot assay. (**A**) Phosphorylation status of ribosomal s6 kinase1 (RSK1), mitogen and stress-activated protein kinase (MSK) , and extracellular signal-regulated kinase (ERK); (**B**) phosphorylation status of p70S6K, AKT, 4e-BP1 and (**C**) investigation of the effect of ERK, RSK, and AKT inhibitor. Band intensity was normalized to GAPDH and values are expressed relative to the control group.

To further investigate the downstreaming signaling pathways of SPARC, the protein was extracted from Detroit 562 after 1.5 h of SPARC treatment. In Figure 5A,B, increased levels of ERK and AKT were observed. The phosphorylation of other proteins was not significantly changed by SPARC treatment. ERK and AKT are important regulators of EMT pathways [18]. Treatment of the ERK inhibitor and AKT inhibitor decreased the expression level of Slug (Figure 5C). Furthermore, the AKT inhibitor, but not ERK inhibitor, suppressed SPARC treatment-induced Slug expression. This suggests that AKT is a key regulator in SPARC-induced EMT signaling pathways.

### 2.5. Evaluation of SPARC Expression in Clinical Samples

**Figure 6:**
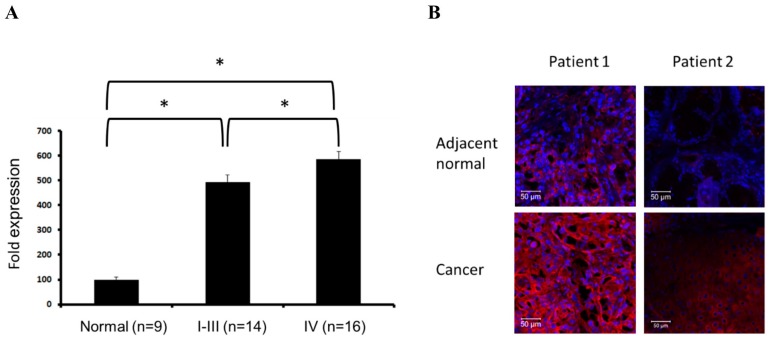
SPARC expression in clinical samples: (**A**) mRNA expression of SPARC in clinical samples. *t*-test; * *p* < 0.05, significant difference between two groups and (**B**) immunofluorescent detection of SPARC in frozen sections of tumors and adjacent normal tissue. Antibody staining is red and nuclei are stained with DAPI. Two of seven pairs are shown. Scale bar = 50 μm.

The expression levels of SPARC in clinical samples were evaluated in a cDNA array assay which included normal tissues and tissues from grade I to IV head and neck cancer patients, including carcinoma of tonsil, pharynx, oropharynx, etc. Our results revealed that mRNA levels of SPARC in normal tissues are significantly lower than that in cancer. In addition, grade IV tumor samples express relatively high SPARC levels in comparison of grade I to III tumor samples (Figure 6A). In four of seven tumor pairs, SPARC expression in tumor region is higher than that in adjacent normal region (Figure 6B). This evidence suggest that high SPARC expression is associated with head and neck tumors in the clinic.

## 3. Discussion

Matricellular proteins are one of important components of extracellular matrix and act at the interface between extracellular matrix and cell surface [7]. SPARC belongs to the matricellular protein family and regulates extracellular matrix assembly [20,21]. Currently, SPARC is reported to be regulated by various signaling molecules. SPARC is required for activation of integrin-linked kinase (ILK) and the ILK-mediated signaling pathways [22]. SPARC induces adipogenesis through activation and accumulation of beta-catenin pathways in preadipocytes [23]. In addition, ILK, but not AKT is required for SPARC-activated beta-catenin pathways [23]. In glioma, AKT and SHC-RAF-ERK signaling are involved in the SPARC-induced invasive capacity [24]. SPARC-expressing glioma cells induce AKT phosphorylation and neutralizing exogenous SPARC by antibodies abolishes the AKT phosphorylation. In addition, exogenous SPARC stimulation results in reducing phosphorylation of AKT in prostate cancer cells [25]. This evidence implies that AKT is a key kinase in SPARC-induced biological process although different phenotypes are regulated in different types of cells.

Clinical evidences have shown that EMT is associated with tumor metastasis and poor prognosis in multiple types of cancers [26]. Transcription factors, such as Slug, Snail, and Twist, downregulate the expression of E-cadherin and Zo-1, and upregulate the expression of *N*-cadherin [18]. In Figure 3, our results showed that SPARC results in increasing levels of *N*-cadherin, Slug, Snail, and Twist, and decreasing levels of E-cadherin in the pharyngeal carcinoma cell line, Detroit 562. However, Zo-1 expression is also induced after treatment. A previous report suggests that Zo-1 did not link to EMT signaling pathways in head and neck squamous cell carcinoma [27]. Thus, our results still reveal SPARC treatment promotes mesenchymal characteristics in Detroit 562.

The present study suggests that AKT is a key molecule in SPARC-induced EMT pathways. Additionally, several soluble factors induce EMT through AKT in different types of head and neck cancer. AKT involves in the platelet-derived growth factor D (PDGF-D) and receptor of PDGF-D-triggered EMT in tongue squamous carcinoma cells [28]. Interleukin (IL)-6 enhances migration and EMT via increasing phosphorylation of p70S6K, and phosphoinositide 3-kinase (PI3K)/AKT/mechanistic target of rapamycin (mTOR), mitogen-activated protein kinase (MAPK)/AKT, and janus kinase (JAK)/STAT3 might be important upstream regulators [29]. Mast cell-secreted IL-8 promotes EMT via AKT and the Slug pathway in human thyroid cancer cells [30]. In hypoxic condition, secretion of IL-11, is induced in anaplastic thyroid carcinoma cells and IL-11 activates PI3K/AKT/glycogen synthase kinase 3 β (GSK3β) and results in EMT phenotype [31]. These studies show that AKT plays a critical role in promoting EMT in head and neck cancers. A recent report demonstrates that blockage of PI3K/AKT signaling pathway attenuate metastasis in nasopharyngeal carcinoma cells [32]. It might imply that depletion of exogenous molecules which activate AKT signaling is a potential strategy for inhibiting metastasis in head and cancer.

**Figure 7:**
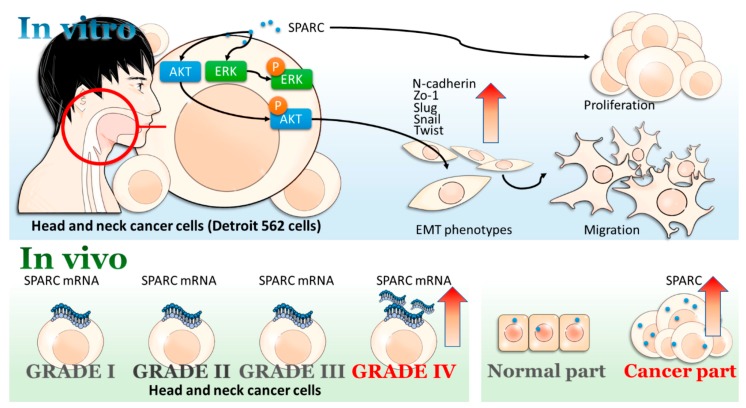
Scheme of proposed SPARC-induced signaling pathways in head and neck cancer.

In the clinical samples, the expression of SPARC in tumor tissues is higher than that in normal tissues and the highest SPARC expression was detected in grade IV tumors (Figure 6). Previous reports suggest that SPARC serves as a prognostic marker for the stage II tongue carcinoma and is positively correlated with lymph node metastasis and survival rate in stage II tongue squamous cell carcinoma [33,34]. In nasopharyngeal carcinoma patients, SPARC expression is also positively correlated with status of distant metastasis, world health organization histological classification, and poor prognosis [35]. The higher SPARC expression is detected in oral squamous cell carcinoma via immunohistochemical analysis when compared with control tissue [36]. Both SPARC in squamous cell carcinoma of the oral cavity and tumor-associated stromal cells are associated with poor prognostic factors, such as daily smoking [37]. In Figure 6B, immunofluorescent analysis showed SPARC expression in tumors is higher than that in adjacent normal tissue. Due to the small sample size, the difference of SPARC expression pattern could not reach statistical significance. Therefore, based on the results of cDNA array and previous reports, SPARC is considered as a risk factor and might serve as a poor prognostic marker of head and neck cancer. In addition, we suppose that the increasing trend of SPARC might be observed in a large sample size. The proposed mechanism was shown in Figure 7.

SPARC shows an anti-tumor role in anti-angiogenesis, anti-adipogenesis, pro-apoptosis, and inhibition of cell proliferation in several types of cancer, including ovarian cancer, colorectal, neuroblastoma [10,38]. These types of cancer has less metastastic capacity when compared to the glioblastomas, melanoma, breast cancer, and prostate cancer. In head and neck cancer, the SPARC-induced effect and signaling pathways tends to a oncogenic role. Additinally, SPARC affects the immune cell infiltration and differentiation [9]. However, the interaction among tumor cells, stroma cells, and immune cells is very complicated. The present study focused on the role of SPARC in tumor cells. The effect of SPARC on stroma cells and immune cells will be evaluated in the further studies.

## 4. Materials and Methods

### 4.1. Cell Culture

The human cancer cell lines, Detroit 562 (pharyngeal carcinoma) and FaDu (which is derived from human hypopharyngeal tumor) were purchased from bioresource collection and research centers (BCRC) of Taiwan. Both cells were cultured in minimum essential medium (MEM) which was supplemented with 10% fetal bovine serum (FBS), 100 g/mL streptomycin, 0.25 mg/mL amphotericin, and 100 unit/mL penicillin (LifeTechnologies, Grand Island, NY, USA) and incubated at 37 °C, in 5% CO_2_.

### 4.2. Chemicals

AKT Inhibitor IV (cat. no. 124011), MEK1/ERK inhibitor PD 98059 (cat. no. 513000), and RSK inhibitor SL0101 (cat. no. 559285) were obtained from Calbiochem (San Diego, CA, USA).

### 4.3. Proliferation Assay

In 9a6 well plate, 3000 of Detroit 562 or FaDu cells were seeded and recombinant human SPARC protein (R&D Systems) was treated 24 h after seeding cells. Cells were cultured at 100 μL minimum essential media with 1% FBS and, respectively, 0, 10, 20, and 50 ng/100 μL recombinant human SPARC protein which was directly dissolved in the culture medium for 72 h. WST-1 (4-[3-(4-iodophenyl)-2-(4-nitrophenyl)-2H-5-tetrazolio]-1,3-benzene disulfonate) method (Clontech) was used for determining cell proliferation according to manufacturer’s instruction. The results were analyzed on a microplate spectrophotometer (PowerWave X340, BioTek, Winooski, VT, USA) at a wavelength 450 nm.

### 4.4. Migration Assay

Migration ability was analyzed by wound-healing assay and transwell migration assay. FaDu cells (4 × 10^5^) and Detroit 562 cells (5 × 10^5^) were seeded into 24 well plates. When cells reached a 90–100% confluent monolayer, a scratch was made by a 200 μL pipette tip. Cell debris was removed by phosphate-buffered saline washing after scratching and then cells were incubated in MEM medium with 2% FBS. Images were taken at 0, 12, 18, and 24 h after SPARC treatment. Transwell migration assay was performed in the QCM™ 24-well Cell Migration Assay and Invasion System uncoated 8 m pore size polycarbonate membranes (Millipore) according to the manufacturer’s instructions. Briefly, 8 × 10^4^ FaDu or 1 × 10^5^ Detroit 562 were seeded into 24 wells and inserted into 300 μL serum-free medium while 500 μL medium with 10% FBS was in the lower chamber. After 24 h, the bottom surface of the membrane was fixed in 10% formaldehyde solution, followed by 0.4 g/L crystal violet staining for 2 h. Cells on the upper surface were removed by a cotton swab after membrane washing. The bottom of the membrane was then visualized using the Olympus inverted microscope at 100× magnification. Four random fields of view were counted and the relative fold of migration in each group was compared to the 0 ng/mL SPARC treated group.

### 4.5. Western Blot

Cells were lysed in radioimmunoprecipitation lysis buffer (RIPA) buffer (Millipore) with protease inhibitor cocktail (Millipore) at a 100:1 ratio on ice for 30 min and the total cell lysate was collected after centrifugation at 4 °C, 12,000× *g* for 15 min. Protein concentration was determined by bicinchoninic acid (BCA) protein assay kit (Thermo Fisher Scientific, Rockford, IL, USA). Equal amount protein was loaded into each lane of 8%–12% sodium dodecyl sulfate polyacrylamide gel electrophoresis (SDS-PAGE) and transferred to polyvinylidene difluoride membranes (Millipore). Membranes were blocked with 5% skim milk in Tris-buffered saline with Tween-20 (TBST) buffer and sequentially incubated with primary antibodies (diluted in 3% FBS in TBST) overnight at 4 °C, and with secondary antibodies for one hour at room temperature. The information and dilution fold is shown: anti-*N*-cadherin (1:1000, cat. no. 2167184, Millipore), anti-E-cadherin (1:1000, cat. no. 610182, BD Biosciences, San Jose, CA, USA), anti-Zo-1 (1:1000, cat. no. 5406S, Cell Signaling, Beverly, MA, USA), anti-Slug (1:1000, cat. no. 9585, Cell Signaling), anti-Snail (1:1000, cat. no.3879, Cell Signaling), anti-Twist (1:1000, cat. no. 49254, Abcam, Cambridge, UK), anti-phospho-ERK 1/2 (1:1000, cat. no. 4370S, Cell Signaling), anti-ERK 1/2 (1:1000, cat. no. 4695S, Cell Signaling), anti-phospho AKT Ser473 (1:1000, cat. no. 4060S, Cell Signaling), anti-phospho AKT Thr308 (1:1000, cat. no. 2965S, Cell Signaling), anti-phospho AKT (1:1000, cat. no. 9272S, Cell Signaling), anti-phospho Msk1 (1:1000, cat. no. 9591S, Cell Signaling), anti-Msk1 (1:1000, cat. no. 3489S, Cell Signaling), anti-phospho Rsk1 (1:1000, cat. no. 9335S, Cell Signaling), anti-Rsk1 (1:1000, cat. no. 8408S, Cell Signaling), anti-phospho p70 S6 (1:1000, cat. no. 9204, Cell Signaling), anti-p70 S6 (1:1000, cat. no. 9202, Cell Signaling), anti-phospho-4E-BP1 (1:1000, cat. no. 2855, Cell Signaling), anti-4E-BP1 (1:1000, cat. no. 9644, Cell Signaling), anti-GAPDH (1:5000, cat. no. MAB374, Millipore). The results were analyzed on Alpha Innotech FluorChem FC2 imaging system (ProteinSimple; Bio-Techne, Minneapolis, MN, USA). The quantification was performed using ImageJ software (version 1.51, National Institutes of Health, Bethesda, MD, USA).

### 4.6. Phospho-Kinase Array

Six-hundred micrograms of protein lysate of Detroit 562 cells were, respectively, treated with or without SPARC for 24 hours were collected. Protein samples were incubated with a Human Phospho-Kinase Array Kit (Proteome Profiler Array, ARY003B, R&D Systems) according to the manufacturer’s instruction. The images were adapted on an imaging capture system (Alpha Innotech FluorChem FC2 imaging system, ProteinSimple; Bio-Techne, Minneapolis, MN, USA).

### 4.7. cDNA Arrays Analysis

The cDNA array of normal tissue and patients with head and neck cancer was obtained from Head and Neck Cancer cDNA Array I (cat. no. HNRT101, TissueScan cDNA array, Origene). Polymerase chain reaction was performed using the following primers: SPARC forward, 5′-GTGCAGAGGAAACCGAAGAG-3′ and reverse, 5′-AGTGGCAGGAAGAGTCGAAG-3′; GAPDH forward, 5′-GAGTCAACGGATTTGGTCGT-3′ and reverse, 5′-TTGATTTTGGAGGGATCTCG-3′. PCR was performed on a StepOne Plus Real-Time PCT System (Applied Biosystems; Thermo Fisher Scientific, Inc.) using the Fast SYBR Green Master Mix (Applied Biosystems; Thermo Fisher Scientific, Inc.). The relative mRNA expression levels were normalized to the expression level of GAPDH using the 2^–∆∆*C*t^ method.

### 4.8. Human Tumor Samples

Seven pairs of tumor and adjacent non-tumor head and neck tissues were collected and admitted to the Division of Plastic and Reconstructive Surgery, Kaohsiung Medical University Hospital (KMUH), Kaohsiung, Taiwan. Approval for these studies was obtained from the Institutional Review Board (IRB) of KMUH (IRB No.: KMUHIRB-E(I)-20170119, all clinical research was approved on 12/05/2017), and informed consent was obtained from all patients in accordance with the Declaration of Helsinki.

### 4.9. Statistical Analysis

All bar graphs and statistics were performed by GraphPad Prism 7 (GraphPad Software, San Diego, CA, USA). Student’s *t*-test and one-way Analysis of variance (ANOVA) were respectively used for analysis of the difference between two groups and more than two groups. A *p*-value < 0.05 was considered to indicate a statistically significant difference.

## 5. Conclusions

SPARC treatment enhances cell proliferation, migration, and mesenchymal phenotype, and AKT is a key molecule in the SPARC-induced EMT signaling pathway. Among the cDNA array assays, the highest SPARC expression is detected in grade IV tumor samples. Thus, the present study suggests that both exogenous SPARC and tumor-expressing SPARC might be associated with head and neck cancers.

## References

[1] Siegel R.L., Miller K.D., Jemal A. (2017). Cancer statistics, 2017. CA Cancer J. Clin..

[2] Rezende T.M., de Souza Freire M., Franco O.L. (2010). Head and neck cancer: Proteomic advances and biomarker achievements. Cancer.

[3] Kreimer A.R., Clifford G.M., Boyle P., Franceschi S. (2005). Human papillomavirus types in head and neck squamous cell carcinomas worldwide: A systematic review. Cancer Epidemiol. Biomarkers Prev..

[4] Gilyoma J.M., Rambau P.F., Masalu N., Kayange N.M., Chalya P.L. (2015). Head and neck cancers: A clinico-pathological profile and management challenges in a resource-limited setting. BMC Res. Notes.

[5] Eckert A.W., Wickenhauser C., Salins P.C., Kappler M., Bukur J., Seliger B. (2016). Clinical relevance of the tumor microenvironment and immune escape of oral squamous cell carcinoma. J. Transl. Med..

[6] Multhaupt H.A., Leitinger B., Gullberg D., Couchman J.R. (2016). Extracellular matrix component signaling in cancer. Adv. Drug Deliv. Rev..

[7] Bornstein P., Sage E.H. (2002). Matricellular proteins: Extracellular modulators of cell function. Curr. Opin. Cell Biol..

[8] Brekken R.A., Sage E.H. (2001). Sparc, a matricellular protein: At the crossroads of cell-matrix communication. Matrix Biol..

[9] Framson P.E., Sage E.H. (2004). Sparc and tumor growth: Where the seed meets the soil?. J. Cell. Biochem..

[10] Feng J., Tang L. (2014). Sparc in tumor pathophysiology and as a potential therapeutic target. Curr. Pharm. Des..

[11] Zhu A., Yuan P., Du F., Hong R., Ding X., Shi X., Fan Y., Wang J., Luo Y., Ma F. (2016). Sparc overexpression in primary tumors correlates with disease recurrence and overall survival in patients with triplenegative breast cancer. Oncotarget.

[12] Nagai M.A., Gerhard R., Fregnani J.H., Nonogaki S., Rierger R.B., Netto M.M., Soares F.A. (2011). Prognostic value of ndrg1 and sparc protein expression in breast cancer patients. Breast Cancer Res. Treat..

[13] Huang Y., Zhang J., Zhao Y.Y., Jiang W., Xue C., Xu F., Zhao H.Y., Zhang Y., Zhao L.P., Hu Z.H. (2012). Sparc expression and prognostic value in non-small cell lung cancer. Chin. J. Cancer.

[14] Beck A.H., Espinosa I., Gilks C.B., van de Rijn M., West R.B. (2008). The fibromatosis signature defines a robust stromal response in breast carcinoma. Lab. Investig..

[15] Guttlein L.N., Benedetti L.G., Fresno C., Spallanzani R.G., Mansilla S.F., Rotondaro C., Raffo Iraolagoitia X.L., Salvatierra E., Bravo A.I., Fernandez E.A. (2017). Predictive outcomes for HER2-enriched cancer using growth and metastasis signatures driven by SPARC. Mol. Cancer Res..

[16] Sato N., Fukushima N., Maehara N., Matsubayashi H., Koopmann J., Su G.H., Hruban R.H., Goggins M. (2003). SPARC/osteonectin is a frequent target for aberrant methylation in pancreatic adenocarcinoma and a mediator of tumor-stromal interactions. Oncogene.

[17] Kalluri R., Weinberg R.A. (2009). The basics of epithelial-mesenchymal transition. J. Clin. Investig..

[18] Lamouille S., Xu J., Derynck R. (2014). Molecular mechanisms of epithelial-mesenchymal transition. Nat. Rev. Mol. Cell Biol..

[19] Polette M., Mestdagt M., Bindels S., Nawrocki-Raby B., Hunziker W., Foidart J.M., Birembaut P., Gilles C. (2007). β-catenin and Zo-1: Shuttle molecules involved in tumor invasion-associated epithelial-mesenchymal transition processes. Cells Tissues Organs.

[20] Giudici C., Raynal N., Wiedemann H., Cabral W.A., Marini J.C., Timpl R., Bachinger H.P., Farndale R.W., Sasaki T., Tenni R. (2008). Mapping of SPARC/BM-40/osteonectin-binding sites on fibrillar collagens. J. Biol. Chem..

[21] Bradshaw A.D. (2009). The role of sparc in extracellular matrix assembly. J. Cell Commun. Signal..

[22] Barker T.H., Baneyx G., Cardo-Vila M., Workman G.A., Weaver M., Menon P.M., Dedhar S., Rempel S.A., Arap W., Pasqualini R. (2005). SPARC regulates extracellular matrix organization through its modulation of integrin-linked kinase activity. J. Biol. Chem..

[23] Nie J., Sage E.H. (2009). SPARC inhibits adipogenesis by its enhancement of β-catenin signaling. J. Biol. Chem..

[24] Thomas S.L., Alam R., Lemke N., Schultz L.R., Gutierrez J.A., Rempel S.A. (2010). Pten augments SPARC suppression of proliferation and inhibits SPARC-induced migration by suppressing SHC-RAF-ERK and AKT signaling. Neuro Oncol..

[25] Shin M., Mizokami A., Kim J., Ofude M., Konaka H., Kadono Y., Kitagawa Y., Miwa S., Kumaki M., Keller E.T. (2013). Exogenous sparc suppresses proliferation and migration of prostate cancer by interacting with integrin β1. Prostate.

[26] Lee J.M., Dedhar S., Kalluri R., Thompson E.W. (2006). The epithelial-mesenchymal transition: New insights in signaling, development, and disease. J. Cell Biol..

[27] Scanlon C.S., Van Tubergen E.A., Inglehart R.C., D’Silva N.J. (2013). Biomarkers of epithelial-mesenchymal transition in squamous cell carcinoma. J. Dent. Res..

[28] Zhang H., Sun J.D., Yan L.J., Zhao X.P. (2016). PDGF-D/PDGFRβ promotes tongue squamous carcinoma cell (TSCC) progression via activating p38/AKT/ERK/EMT signal pathway. Biochem. Biophys. Res. Commun..

[29] Wu D., Cheng J., Sun G., Wu S., Li M., Gao Z., Zhai S., Li P., Su D., Wang X. (2016). P70s6k promotes IL-6-induced epithelial-mesenchymal transition and metastasis of head and neck squamous cell carcinoma. Oncotarget.

[30] Visciano C., Liotti F., Prevete N., Cali G., Franco R., Collina F., de Paulis A., Marone G., Santoro M., Melillo R.M. (2015). Mast cells induce epithelial-to-mesenchymal transition and stem cell features in human thyroid cancer cells through an IL-8-AKT-Slug pathway. Oncogene.

[31] Zhong Z., Hu Z., Jiang Y., Sun R., Chen X., Chu H., Zeng M., Sun C. (2016). Interleukin-11 promotes epithelial-mesenchymal transition in anaplastic thyroid carcinoma cells through PI3K/AKT/GSK3β signaling pathway activation. Oncotarget.

[32] Jiang H., Gao M., Shen Z., Luo B., Li R., Jiang X., Ding R., Ha Y., Wang Z., Jie W. (2014). Blocking PI3K/AKT signaling attenuates metastasis of nasopharyngeal carcinoma cells through induction of mesenchymal-epithelial reverting transition. Oncol. Rep..

[33] Kato Y., Nagashima Y., Baba Y., Kawano T., Furukawa M., Kubota A., Yanoma S., Imagawa-Ishiguro Y., Satake K., Taguchi T. (2005). Expression of sparc in tongue carcinoma of stage II is associated with poor prognosis: An immunohistochemical study of 86 cases. Int. J. Mol. Med..

[34] Zhang J., Zhang Q., Zhang Q., Liu X.K., Li C.Q., Guo Z.M. (2009). Expression and clinical significance of SPARC in clinical stage II tongue squamous cell carcinoma. Chin. J. Cancer.

[35] Wang H.Y., Li Y.Y., Shao Q., Hou J.H., Wang F., Cai M.B., Zeng Y.X., Shao J.Y. (2012). Secreted protein acidic and rich in cysteine (SPARC) is associated with nasopharyngeal carcinoma metastasis and poor prognosis. J. Transl. Med..

[36] Choi P., Jordan C.D., Mendez E., Houck J., Yueh B., Farwell D.G., Futran N., Chen C. (2008). Examination of oral cancer biomarkers by tissue microarray analysis. Arch. Otolaryngol. Head Neck Surg..

[37] Aquino G., Sabatino R., Cantile M., Aversa C., Ionna F., Botti G., La Mantia E., Collina F., Malzone G., Pannone G. (2013). Expression analysis of SPARC/osteonectin in oral squamous cell carcinoma patients: From saliva to surgical specimen. Biomed. Res. Int..

[38] Nagaraju G.P., Sharma D. (2011). Anti-cancer role of sparc, an inhibitor of adipogenesis. Cancer Treat. Rev..

