# Correction: Chang, C.-H.; et al. Secreted Protein Acidic and Rich in Cysteine (SPARC) Enhances Cell Proliferation, Migration, and Epithelial Mesenchymal Transition, and SPARC Expression Is Associated with Tumor Grade in Head and Neck Cancer. *Int. J. Mol. Sci.* 2017, *18*, 1556

**DOI:** 10.3390/ijms19082338

**Published:** 2018-08-09

**Authors:** Chih-Hau Chang, Meng-Chi Yen, Ssu-Hui Liao, Yu-Ling Hsu, Chung-Sheng Lai, Kao-Ping Chang, Ya-Ling Hsu

**Affiliations:** 1 Graduate Institute of Medicine, College of Medicine, Kaohsiung Medical University, Kaohsiung 807, Taiwan; igor8301023@gmail.com (C.-H.C.); s0970215575@gmail.com (S.-H.L.); l32861wa@yahoo.com.tw (Y.-L.H.); 2 Division of Plastic and Reconstructive Surgery, Kaohsiung Medical University Hospital, Kaohsiung 807, Taiwan; chshla@kmu.edu.tw (C.-S.L.); kapich@kmu.edu.tw (K.-P.C.); 3 Department of Emergency Medicine, Kaohsiung Medical University Hospital, Kaohsiung Medical University, Kaohsiung 807, Taiwan; yohoco@gmail.com; 4 Faculty of Medicine, College of Medicine, Kaohsiung Medical University, Kaohsiung 807, Taiwan

We would like to submit the correction to our published paper [1]. The reason for the correction is that the pictures provided in Figure 2A,C were represented by the same picture. Figure 2A is correct (FaDu cell), but Figure 2C is wrong (Detroit 562). For such reason, the Figure 2C should be replaced with the correct picture. The quantitative results provided in Figure 2D is correct. These changes do not change on the results and conclusions of our paper. We apologize for any inconvenience caused.


